# Rapid Spread of Severe Fever with Thrombocytopenia Syndrome Virus by Parthenogenetic Asian Longhorned Ticks

**DOI:** 10.3201/eid2802.211532

**Published:** 2022-02

**Authors:** Xing Zhang, Chaoyue Zhao, Chaoyuan Cheng, Guogang Zhang, Tao Yu, Kevin Lawrence, Hongyue Li, Jimin Sun, Zeyu Yang, Ling Ye, Hongliang Chu, Ying Wang, Xiaohu Han, Yongchao Jia, Shuozhang Fan, Hirotaka Kanuka, Tetsuya Tanaka, Cheryl Jenkins, Kristene Gedye, Shona Chandra, Dana C. Price, Qiyong Liu, Young Ki Choi, Xiangjiang Zhan, Zhibin Zhang, Aihua Zheng

**Affiliations:** Chinese Academy of Sciences, Beijing, China (X. Zhang, C. Zhao, C. Cheng, H. Li, X. Zhan, Z. Zhang, A. Zheng);; University of Chinese Academy of Sciences, Beijing (X. Zhang, C. Zhao, C. Cheng, H. Li, Z. Zhang, A. Zheng);; Yantai Center for Disease Control and Prevention, Yantai, China (T. Yu);; Massey University, Palmerston North, New Zealand (K. Lawrence, K. Gedye);; Zhejiang Center for Disease Control and Prevention, Zhejiang, China (J. Sun);; Chinese Academy of Forestry, Beijing (Z. Yang, G. Zhang);; Daishan Center for Disease Control and Prevention, Zhoushan, China (L. Ye);; Jiangsu Center for Disease Control and Prevention, Nanjing, China (H. Chu);; Xinyang Center for Disease Control and Prevention, Xinyang, China (Y. Wang);; Shenyang Agricultural University, Shenyang, China (X. Han);; Guangyuan Center for Disease Control and Prevention, Guangyuan, China (Y. Jia);; Hebei University, Baoding, China (S. Fan);; Jikei University School of Medicine, Tokyo, Japan (H. Kanuka);; Kagoshima University, Kagoshima, Japan (T. Tanaka);; Elizabeth Macarthur Agricultural Institute, Menangle, New South Wales, Australia (C. Jenkins);; University of Sydney, Camden, New South Wales, Australia (S. Chandra);; Rutgers University, New Brunswick, New Jersey, USA (D.C. Price):; National Institute for Communicable Disease Control and Prevention, Beijing (Q. Liu);; Chungbuk National University, Cheongju City, South Korea (Y.K. Choi)

**Keywords:** severe fever with thrombocytopenia syndrome virus, SFTSV, severe fever with thrombocytopenia syndrome, SFTS, ticks, Asian longhorned ticks, parthenogenetic ticks, bisexual ticks, spread, vector-borne infections, migratory birds, zoonoses, China

## Abstract

Severe fever with thrombocytopenia syndrome virus (SFTSV) is spreading rapidly in Asia. This virus is transmitted by the Asian longhorned tick (*Haemaphysalis longicornis*), which has parthenogenetically and sexually reproducing populations. Parthenogenetic populations were found in ≥15 provinces in China and strongly correlated with the distribution of severe fever with thrombocytopenia syndrome cases. However, distribution of these cases was poorly correlated with the distribution of populations of bisexual ticks. Phylogeographic analysis suggested that the parthenogenetic population spread much faster than bisexual population because colonization is independent of sexual reproduction. A higher proportion of parthenogenetic ticks was collected from migratory birds captured at an SFTSV-endemic area, implicating the contribution to the long-range movement of these ticks in China. The SFTSV susceptibility of parthenogenetic females was similar to that of bisexual females under laboratory conditions. These results suggest that parthenogenetic Asian longhorned ticks, probably transported by migratory birds, play a major role in the rapid spread of SFTSV.

Parthenogenesis is the development of an embryo from an unfertilized egg and is a common reproductive mechanism in invertebrate arthropods, especially insects and mites (*1*). One of the potential advantages of parthenogenesis is that the offspring are all genetically identical, which in relatively stable environments can lead to a rapid expansion in numbers. After dispersal, new populations can be established from 1 female. The Asian longhorned tick (*Haemaphysalis longicornis*) is among a small number of medically relevant tick species that have parthenogenetic and bisexual populations (*2*). The parthenogenetic population of these ticks originated in northern Japan (*3*) and is now common in the Asia‒Pacific region. Parthenogenetic and bisexual populations are found in eastern East Asia, but only the parthenogenetic population is found in Oceania (*3*). In China, the parthenogenetic population has only been reported in a few locations (*4*). During 2017, parthenogenetic Asian longhorned ticks were found in New Jersey, USA (*5*), and by 2020, they had been found across 12 states, primarily in the eastern United States (*6*).

Severe fever with thrombocytopenia syndrome virus (SFTSV) is a tickborne phlebovirus transmitted by Asian longhorned ticks that was described in China during 2009 at the border of Henan, Anhui, and Hubei Provinces (*7*,*8*). SFTSV is maintained and transmitted by Asian longhorned ticks in the larva, nymph, and adult stages in both transovarial and transstadial modes (*8*–*10*). Human mortality rates for SFTSV infection range from 6% to 30% (*7*,*11*). During 2011‒2016, cases of severe fever with thrombocytopenia syndrome (SFTS) were reported in 18 of the 34 provinces in China, and there was a 3-fold increase in case number (from 500 cases to 1,500 cases) (*11*). Most cases were in the rural areas of Henan (37%), Shandong (26.6%), Anhui (14%), and Hubei (12.6%) Provinces (*11*). SFTSV has also been reported in South Korea (2012), Japan (2014), Vietnam (2019), and Pakistan (2020) (*12*–*15*). Phylogenetic analysis showed that SFTSV isolates separate into the Chinese clade and the Japanese clade, which is consistent with their geographic distribution (*16*). A close relative of SFTSV, Heartland virus, was reported in the United States during 2012 and is transmitted by *Amblyomma americanum* ticks (*17*,*18*).

The Asian longhorned tick is the dominant tick species in SFTSV-endemic areas. Tick prevalence rates are 88% in Jiangsu Province, China, and 91% in Gangwon Province, South Korea (*19*,*20*). Most of the SFTSV-endemic areas are rural, and 97% of the patients are farmers living in wooded and hilly areas, far from modern transportation and cities (*21*). The rapid spread of SFTSV is unexplained, although Asian longhorned ticks have a broad host range (*18*), enabling several possible modes of dissemination. For example, because livestock and wild mammals are common hosts for Asian longhorned ticks, grazing of cattle or foraging of wild mammals, such as hares, could rapidly distribute ticks in an area with a suitable habitat (*22*). Furthermore, birds range far enough to transport ticks within a district, and long-range dispersal of tickborne pathogens can also be accelerated by tick-infested migratory birds (*23*).

During 2016, SFTSV antibodies were detected in tick-infested migratory greater white-fronted geese in Jiangsu Province, China (*24*). This finding has led to the suggestion that migratory birds might have been involved in the rapid spread of this disease in China. Two areas of China that have high endemic levels of SFTSV are situated on major bird migratory routes (*25*). The Dabie Mountain area region, where SFTSV was originally reported, is in the middle of a major bird migration route from Dongting Lake and Poyang Lake to Siberia, and Penglai City and Dalian City are located on the northern part of the Asia‒Pacific migratory route. Dongting Lake and Poyang Lake are 2 of the major overwintering sites for migratory birds in China (*26*,*27*).

The aim of this study was to test the hypothesis that parthenogenetic Asian longhorned ticks, possibly carried by migratory birds, are responsible for the rapid spread of SFTSV in China and Asia. To test this hypothesis, we conducted a series of linked experiments, which included mapping the distribution of bisexual and parthenogenetic Asian longhorned ticks in those provinces in China that had a high prevalence of SFTS. We also estimated the geographic correlation between bisexual and parthenogenetic ticks and SFTS cases for those provinces, surveyed infestation of Asian longhorned ticks in migratory birds in a city that had a high prevalence of SFTS, tested virus acquisition and transstadial passage of Asian longhorned ticks for SFTSV, inferred the phylogeny of Asian longhorned ticks by using ticks collected from other regions, and estimated the correlation between migratory bird routes and bisexual and parthenogenetic tick populations in China.

## Materials and Methods

### Ethics

We conducted all animal studies in accordance with the recommendations in the Guide for the Care and Use of Laboratory Animals of the Ministry of Science and Technology of the People’s Republic of China. Protocols for animal studies were approved by the Committee on the Ethics of Animal Experiments of the Institute of Zoology, Chinese Academy of Sciences (approval no. IOZ-IACUC-2020-062).

### Tick Collection in China

**Figure 1:**
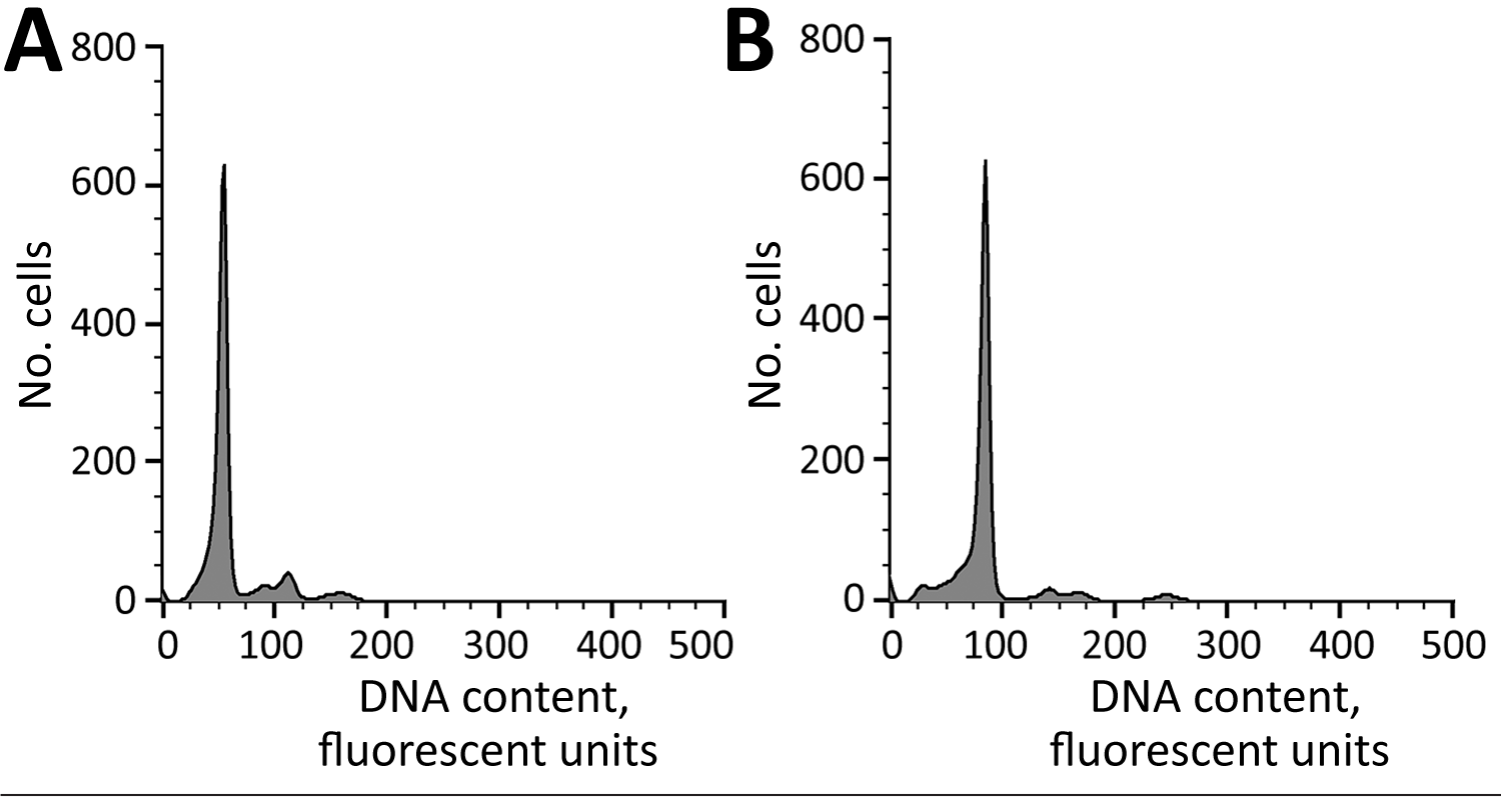
Polyploid analysis of bisexual and parthenogenetic Asian longhorned tick populations in China. Ploidy of ticks was tested by using flow cytometry and measuring fluorescence intensity of cell nuclei stained with 4′,6-diamidino-2-phenylindole. A) Bisexual (2n) sample with a peak at the 66 position. B) Parthenogenetic (3n) sample with a peak at the 99 position.

We collected Asian longhorned ticks from 73 counties covering 20 provinces in China to which SFTSV is endemic (Figure 1; Appendix Table 1). We collected ticks of all life stages by using flag-dragging and removal directly from animals during April‒November 2019. Were identified ticks on the basis of morphologic characteristics, visualized through a light microscope, and further confirmed by molecular analysis in the laboratory by sequencing the mitochondrial 16S rRNA gene by using primers 16S-1, 5′-CTGCTCAATGATTTTTTAAATTGCTGTGG-3′, and 16S-2, 5′-CGCTGTTATCCCTAGAGTATT-3′. We removed 1 leg from each tick for molecular analysis to confirm identification. We used a random sample of 5‒6 live ticks from each county sampled and stored then at room temperature until ploidy detection; we stored the remaining tick specimens at −80°C.

### Tick Collection in Other Regions

We obtained 8 extracted tick DNA samples from overseas collaborators, including those in Japan, South Korea, New Zealand, Australia, and the United States during 2019 (Appendix Table 2). We subjected these ticks to the same molecular analysis as the Asian longhorned ticks collected in China.

### Polyploid Analysis of Ticks

Because bisexual and parthenogenetic ticks are difficult to distinguish by using classical taxonomic methods but can be identified by karyotype analysis, we used flow cytometry to test the ploidy of tick chromosomes (*4*). This identification was accomplished by measuring the fluorescence intensity of cell nuclei stained with 4′,6-diamidino-2-phenylindole. We used the Sysmex Partec CyFlow Apparatus Space (Sysmex Partec, https://www.sysmex-partec.com) in this analysis and the Sysmex Partec CyStain DNA 1 Step Kit.

### Correlation between Bisexual and Parthenogenic Ticks and Cases of SFTS

We analyzed the geographic correlation between SFTS cases and the distribution of different populations of Asian longhorned ticks by using linear regression (*28*). We used the total number of recovered ticks, aggregated at the province or municipality level, as the independent variable and the incidence of SFTS cases (cases per million persons) reported in each respective province or municipality as the dependent variable. We obtained data for SFTS cases summarized by province or municipality for 2019 from the Chinese Center for Disease Control and Prevention.

### Phylogenetic Tree and Genetic Distance

For each county, we randomly picked 1 bisexual tick sample or 1 parthenogenetic tick sample for whole mitochondrial sequencing. We sequenced whole mitochondrial genomes by using next-generation sequencing (Tsingke Biotech, https://career.tsinghua.edu.cn). We performed phylogenetic analysis by using whole mitochondrial genomes of 46 bisexual and 35 parthenogenetic ticks collected from China, Japan, South Korea, New Zealand, Australia, and the United States. We extracted Tick DNA by using the MightyPrep Reagent for DNA Kit (TaKaRa, https://www.takarabio.com) according to the manufacturer’s instructions. We sequenced mitochondrial DNA by using next-generation sequencing (Tsingke Biotech) and deposited sequences in GenBank (accession nos. MW642336‒407). We constructed 2 phylogenetic trees by using this data. the first tree by using the maximum-likelihood method MEGA-X (https://www.megasoftware.net) with the bootstrap value set at 1,000 and the second tree by using the Bayesian interference method with MrBayes version 3.2.7 (http://nbisweden.github.io/MrBayes/index.html) and 1,500,000 generations. We used the genetic distance (GD) to calculate the dispersal index, which was equal to the nucleotide substitution rate.

### Nucleotide Diversity

**Figure 8:**
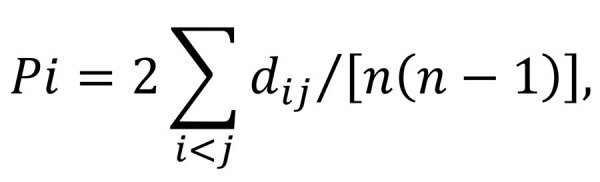
Equation

The nucleotide diversity (Pi) in each dataset is estimated by the equation (Figure 8)

where *d_ij_* is an estimate of the number of nucleotide substitutions per site between DNA sequences *i* and *j* and *n* is the number of DNA sequences examined (*29*). We implanted the calculation for *Pi* in DnaSP-V6 (*30*).

### Dispersal Index

Because without longitudinal surveillance data, we could not evaluate the spread velocity of ticks directly, we defined a dispersal index to quantify and compare the dispersal ability of bisexual and parthenogenetic ticks. According to the molecular clock theory, because the mutation rate is relatively constant, genetic distance can be used to represent time distance (*31*). The dispersal index (I) was defined as I = D/G, where D represents the sum of the spatial distances between every 2 samples (i.e., pairwise geographic distance) and G represents the genetic distance (nucleotide substitutions) between every 2 samples (i.e., pairwise genetic distance). This index is not the true dispersal velocity itself, but we can infer that the greater the dispersal index value, the greater the dispersal velocity. We performed an independent sample *t*-test to contrast the dispersal rates for bisexual and parthenogenetic ticks. We calculated dispersal index and *t*-test by using customed scripts written in Python version 3.7 (https://www.python.org).

### Migratory Bird Capture and Tick Collection

To test our hypothesis that migratory birds are carrying Asian longhorned ticks, we investigated the presence of ticks on northward migratory birds at a high-density stopover site at Penglai City (37°55′13.84″N, 120°43′53.27″E), Yantai City, Shandong Province, during April 2021. Shandong Province is a major location on the Asia‒Pacific migratory route. We captured birds by using mist nets (2.5 × 6 m or 2.5 × 12 m, mesh size 3.0 cm) placed in a wooded habitat. Upon capture, we meticulously examined birds for ticks by searching ear canals, backs of heads, mandibular areas, and perimeter of the eyes. We then removed ticks by using fine forceps.

### Tick Diversity in Penglai City

Penglai City is 1 of the most SFTS-endemic areas in China and a major location in the Asia‒Pacific migratory route. We collected and sequenced parthenogenetic ticks from 9 locations in Penglai City. We used phylogenetic analysis to compare the diversity of ticks from Penglai City with the diversity of ticks from 15 other provinces in China.

### Virus Acquisition by and Transstadial Passage of Ticks for SFTSV

We tested the virus susceptibility of parthenogenetic and bisexual populations for transmitting SFTSV by using laboratory-adapted Asian longhorned tick colonies and an IFNAR (interferon α/β receptor knockout) mouse model. We infected nymphal ticks by feeding them on IFNAR^−/−^ C57/BL6 mice, which were previously inoculated with 2 × 10^3^ focal-forming units of SFTSV (Wuhan strain; GenBank accession nos.: small segment, KU361341.1; medium segment KU361342.1; large segment, KU361343.1). We collected fed nymphs after they were fully engorged and had detached from the mice. We analyzed SFTSV RNA levels in the ticks after they molted into adults. We extracted total RNA prepared from homogenates of ticks by using TRIzol reagent (Thermo Fisher Scientific, https://www.thermofisher.com) according to manufacturer’s instructions. We analyzed samples by using a One-Step SYBR PrimerScript Reverse Transcription PCR Kit (TaKaRa) on an Applied Biosystems QuantStudio (https://www.thermofisher.com) and measured each sample in triplicate. Primers were designed as previously described (*32*).

## Results

### Tick Distribution and Ploidy Analysis

**Figure 2:**
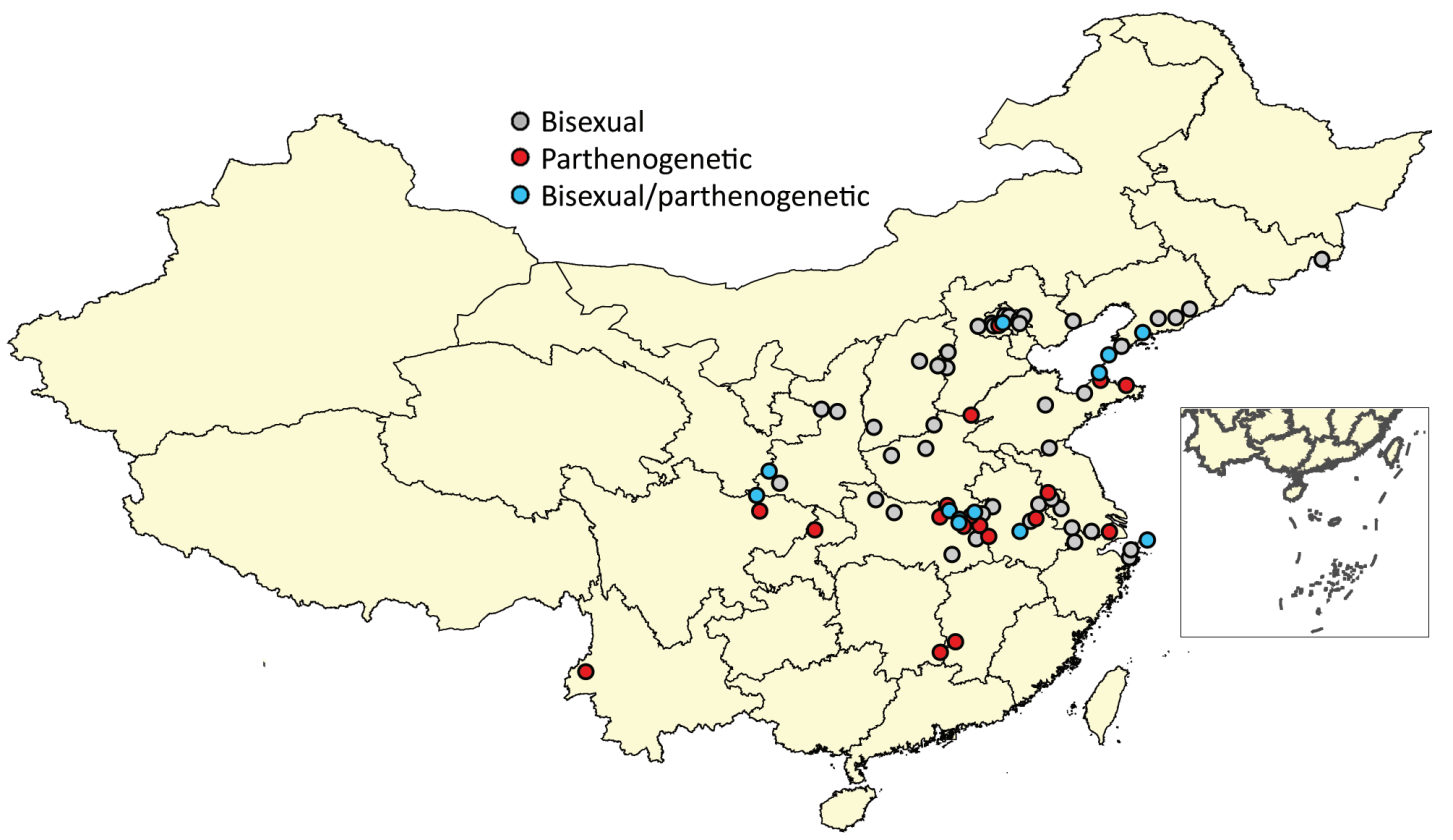
Geographic distribution of bisexual and parthenogenetic Asian longhorned ticks collected in China. Red dots indicate parthenogenetic ticks, gray dots indicate bisexual ticks, and blue dots indicate bisexual and parthenogenetic ticks.

There were 1,328 Asian longhorned ticks confirmed by 16S rRNA sequencing, of which 271 (20.4%) live ticks were further submitted for ploidy analysis by flow cytometry (255 ticks) or by mitochondrial sequencing (16 ticks) (Appendix Table 1). Ploidy testing showed a peak for the bisexual (diploid) population at the 66 position and for the parthenogenetic (triploid) population at the 99 position (Figure 1). Of ticks submitted for ploidy analysis, 186 (69%) of 271 were identified as bisexual and 85 (31%) of 271 as parthenogenetic. Bisexual ticks were detected in 55 (75%) of 73 counties, parthenogenetic ticks were detected in 30 (42%) of 73 of counties, and a mixture of both populations was detected in 12 (16%) of 73 counties (Figure 2; Appendix Table 1). In 18 (25%) of 73 counties, only parthenogenetic ticks were found, and in 43 (59%) of 73 counties, only bisexual ticks were found.

### Correlation of SFTSV with Bisexual and Parthenogenic Ticks

**Figure 3:**
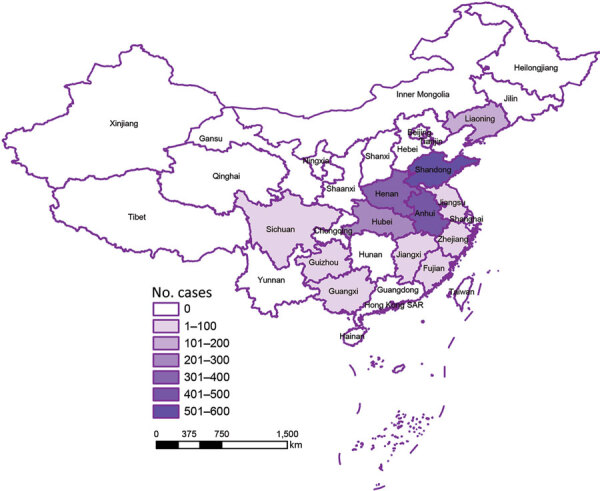
Distribution of severe fever with thrombocytopenia syndrome cases in China during 2019 (Chinese Center for Disease Control and Prevention), showing high correlation with parthenogenetic Asian longhorned tick population (shown in Figure 2).

**Figure 4:**
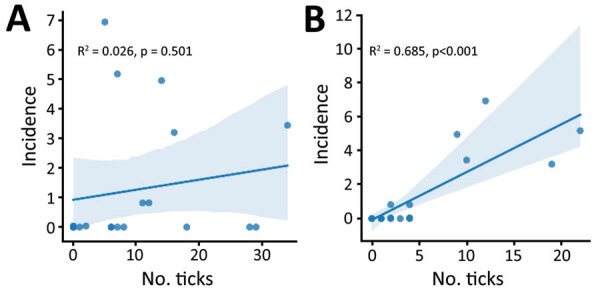
Correlation between incidence of severe fever with thrombocytopenia syndrome virus cases (cases per 1 million persons) and number of bisexual (A) and parthenogenetic (B) Asian longhorned ticks in different provinces, China. Each dot represents the number of cases in a province. Blue shading indicates 95% CI.

SFTS cases showed a strong correlation with the parthenogenetic population (*R^2^* = 0.685, p<0.001) but almost no correlation with the bisexual population (*R^2^* = 0.026, p = 0.501) (Figure 3, Figure 4). In the highly endemic Dabie Mountain area (located at the border of Henan, Anhui, and Hubei Provinces in central China), 66% of the collected Asian longhorned ticks were parthenogenetic in 11 of 14 counties (Appendix Table 1). These results suggest that the parthenogenetic populations of Asian longhorned ticks are strongly associated geographically with cases of SFTS.

### Phylogenetic Analysis of Bisexual and Parthenogenic Ticks

**Figure 5:**
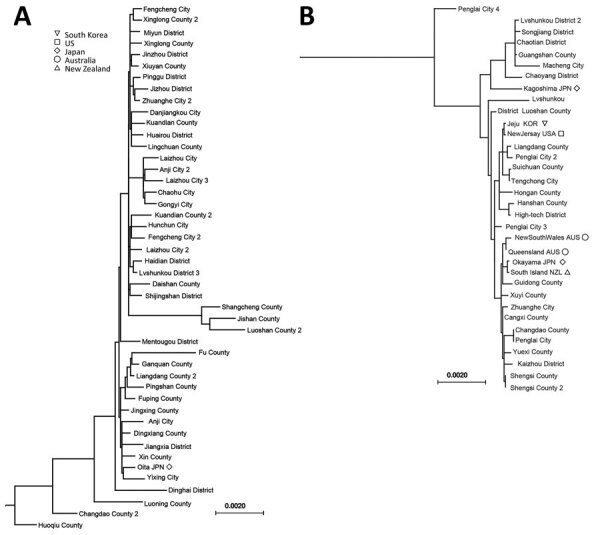
Phylogenetic analysis of bisexual (A) and parthenogenetic (B) Asian longhorned ticks in China and other countries. Maximum-likelihood trees were established with mitochondrial genomes of ticks collected in the Asian‒Pacific region. Numbers indicate multiple Asian longhorned ticks from the same county. Scale bars indicate nucleotide substitutions per site.

For each county, 1 bisexual or parthenogenetic Asian longhorned tick was submitted for mitochondrial sequencing. We obtained 81 whole mitochondrial genomes from 73 ticks from China ticks and 8 ticks from outside China (Appendix Table 2). Results clearly show that the parthenogenetic and bisexual populations are divided into 2 distinct lineages that can be discriminated by 1 T deletion at position 8497 in the untranslated region (Figure 5; Appendix Figures 2‒5). This finding suggests that the parthenogenetic population might have originated from 1 event without gene exchange. The mean GD between all sequences was 0.0078, as measured by the nucleotide substitution rate. The parthenogenetic strains from New Zealand and Australia were similar to the parthenogenetic strain from Okayama, Japan (mean GD 0.0003). The parthenogenetic strain from Kagoshima, Japan, was a close relative to strains collected from Beijing, Hubei, and Henan, China (Appendix Figure 1), which are geographically separate. The strain from New Jersey, USA, was similar to the strain from Jeju Island, South Korea (GD 0.0001).

### Genetic Diversity

Despite the loss of sexual reproduction, high genetic diversity has been reported in the asexual populations of many insect species (*32*). The Pi values for the 2 populations, as measured by the mitochondrial genome, were 0.00249 for bisexual and 0.00188 for parthenogenetic. These results indicate that the genetic diversity of the bisexual and parthenogenetic populations was similar and that the parthenogenetic population might have diverged from the bisexual population at an early age.

### Dispersal Index of Bisexual and Parthenogenic Ticks

**Figure 6:**
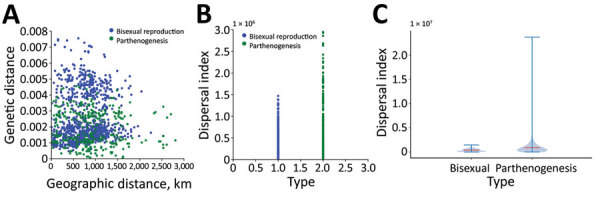
Phylogeographic analysis of bisexual and parthenogenetic Asian longhorned ticks, China. A) Distribution of bisexual and parthenogenetic Asian longhorned ticks in pairwise genetic distance and pairwise geographic distance. B, C) Distribution (B) and difference (C) of dispersal index between bisexual and parthenogenetic Asian longhorned ticks. Horizontal red line in the violin plot indicates the mean dispersal index, shaded blue areas indicate the kernel density estimation, and error bars indicate the maximum (top line) and minimum (bottom line) values.

When compared with bisexual ticks, we found that parthenogenetic ticks have a wider pairwise geographic distance distribution and a narrower pairwise genetic distance distribution (Figure 6, panel A). The dispersal index for parthenogenetic ticks was significantly higher than that for bisexual ticks (*t* = 7.67, p<0.001), and the mean dispersal index for parthenogenetic ticks (910,228) was 2.3 times higher than that for bisexual ticks (393,156) (Figure 6, panels B, C). These results indicate that parthenogenetic ticks have a higher dispersal capacity.

### Correlation between Migratory Birds and Ticks

We collected and examined migratory birds for Asian longhorned ticks in Penglai City, which is an area to which SFTSV is highly endemic and is located in the Asia‒Pacific migratory route (Appendix Figure 1). We netted 95 birds in 17 species. However, 54 Asian longhorned ticks were found on only 4 species: Naumann's thrush (*Turdus naumanni*), grey-backed thrush (*Turdus hortulorum*), great tit (*Parus major*), and chestnut-eared bunting (*Emberiza fucata*). Only 27 ticks were recovered from these birds, of which 19 (70%) were identified as Asian longhorned ticks; 17 (89%) of 19 Asian longhorned ticks were parthenogenetic (Table). All recovered Asian longhorned ticks were nymphs.

**Table:** *Haemaphysalis longicornis* ticks collected from migratory birds and their hosts in Penglai City, China, 2021

Avian host	No. birds examined	No. birds with ticks	No. ticks	No. Asian longhorned ticks	Parthenogenetic,%
*Turdus naumanni*	45	8	11	3	33
*Turdus hortulorum*	7	2	8	8	100
*Parus major*	1	1	5	5	100
*Emberiza fucata*	1	1	3	3	100

### Tick Diversity in Penglai City

Phylogenetic analysis showed that the mitochondrial sequences of the parthenogenetic Asian longhorned ticks collected in Penglai City from vegetation were highly diverse when compared with those from 15 provinces in China (Appendix Figure 6). These data suggest that ticks from many different provinces were present in Penglai City and were probably spread to this region by migratory birds.

### Virus Acquisition by Ticks and Transstadial Passage for Spreading SFTSV

**Figure 7:**
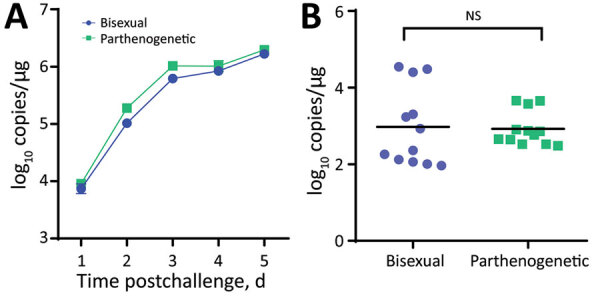
Susceptibility of bisexual and parthenogenetic Asian longhorned ticks to severe fever with thrombocytopenia syndrome virus (SFTSV), China. Groups of bisexual or parthenogenetic nymph Asian longhorned ticks were fed separately on 1 IFNAR^−/−^ (interferon α/β receptor knockout) C57/BL6 mouse that was intraperitoneal inoculated with 2 x 10^3^ focus-forming units of SFTSV. A) Viremias of IFNAR^−/−^ C57/BL6 mice were monitored by using real-time PCR during tick feeding. B) SFTSV infection in the Asian longhorned ticks were tested by real-time PCR after molting into adults. Each dot or square indicates 1 tick. Black horizontal bars indicate means. NS, not significant.

We detected a robust viremia in mice inoculated with SFTSV (Figure 7, panel A). After feeding until engorgement and molting, the parthenogenetic and bisexual populations showed average titers of 3 log RNA copies/mg without obvious differences (Figure 7, panel B). The SFTSV-acquisition and transstadial passage efficiency of the parthenogenetic population appeared comparable with that of the bisexual population.

## Discussion

We found that the parthenogenetic population of Asian longhorned ticks is more widely distributed in China than previously believed and that the distribution is highly correlated with regions to which SFTSV is endemic. Phylogeographic analysis suggests that the parthenogenetic Asian longhorned tick population has spread more rapidly over a greater distance than the bisexual population, and assessment of virus acquisition and transstadial passage showed that bisexual and parthenogenetic populations were comparable in maintaining local transmission of SFTSV. Although only a small number of ticks were recovered, parthenogenetic Asian longhorned ticks were the dominant variety found in migratory birds collected in an area to which SFTS is endemic. We suggest that these results strongly support the hypothesis that parthenogenetic Asian longhorned tick populations are responsible for the rapid spread of SFTSV within China, most likely through being disseminated by migratory birds.

If, as we suggest, migratory birds have played a major role in the spread of parthenogenetic Asian longhorned ticks, then this role would partially explain the wide distribution of these ticks from the cold, far eastern region of Russia to the tropical areas of Australia and the Fiji Islands. However, the role of livestock, wild mammals, companion animals, and humans in translocation of parthenogenetic Asian longhorned ticks should not be overlooked (*22*).

Migratory birds are known to be carriers of ticks. Penglai City is 1 of the most endemic areas for SFTS and is a key passage in the northern part of East Asian‒Australasian Flyway. In this area, 96% of Asian longhorned ticks were parthenogenetic and showed extremely high diversity (Appendix Figure 6). During a spring bird survey in Penglai City during 2021, we found that Asian longhorned ticks were found in 4 bird species (*Turdus naumanni*, *Turdus hortulorum*, *Parus major*, and *Emberiza fucata*), and 89% of them were parthenogenetic. Among the 4 bird species, 3 of them (*Turdus naumanni*, *Turdus hortulorum*, and *Emberiza fucata*) migrate between eastern Asia and Siberia, and are occasionally found in Alaska (https://www.ebird.org). The preferred habitats for these 4 species are grasslands and bushes, which are also the preferred habitats of Asian longhorned ticks. These results suggest that migratory birds have a major role in long-range movement of parthenogenetic ticks within China and potentially even transoceanic spread of SFTSV.

Parthenogenetic Asian longhorned ticks are also implicated in the spread of a pathogenic form of the blood parasite *Theileria orientalis* throughout the Asia‒Pacific region (*18*). Asian longhorned ticks are purported to have been introduced to Australia in the 19th century from northern Japan and later disseminated to New Zealand, New Caledonia, and Fiji. This theory is supported by phylogenetic results of this study, which show that the New Zealand and Australia Asian longhorned ticks are alike and closely resemble the parthenogenetic strain from Okayama, Japan (*33*). *T. orientalis* parasites have been present in Australia for >100 years, having been introduced with the vector tick, and until 2006 caused only minor signs in livestock (*34*). During 2006, the pathogenic Ikeda genotype of *T. orientalis* was introduced from eastern Asia into New South Wales, Australia (*35*) and by 2014, had spread to most of the states in Australia (*36*). The recent spread of *T. orientalis* parasites across the Asia‒Pacific region and into North America highlights the risk for rapid disease agent transmission into areas in which a competent vector (Asian longhorned tick) is already established. Thus, although SFTSV has not yet been detected in the Western Hemisphere, the presence of Asian longhorned parthenogenetic ticks in several countries within the study region presents a clear risk for future emergence of this virus.

Appendix Additional information on infection of parthenogenetic Asian longhorned ticks with severe fever with thrombocytopenia syndrome virus.
