# Supplementary material for: Rapid Spread of Severe Fever with Thrombocytopenia Syndrome Virus by Parthenogenetic Asian Longhorned Ticks
Source: Emerg Infect Dis. 2022 Feb;28(2):363–72. doi: 10.3201/eid2802.211532 (PMC8798674; doi:10.3201/eid2802.211532)
Supplement: Appendix — Additional information on infection of parthenogenetic Asian longhorned ticks with severe fever with thrombocytopenia syndrome virus. [file 21-1532-Techapp-s1.pdf]

Article DOI: https://doi.org/10.3201/eid2802.211352

Rapid Spread of Severe Fever with Thrombocytopenia Syndrome Virus by Parthenogenetic Asian Longhorned Ticks

Appendix

Appendix Table 1. Location and polyploid information for Asian longhorned tick samples, China

Location	No. ticks identified	No. Asian longhorned ticks identified	No. bisexual Asian longhorned ticks	No. parthenogentic Asian longhorned ticks
Yuexi County, Anqing City, Anhui Province*	3	3	0	3
Nanqiao District, Chuzhou City, Anhui Province	24	1	1	0
Chaohu City, Hefei City, Anhui Province	2	2	2	0
Lujiang County, Hefei City, Anhui Province*†	10	7	1	6
Huoqiu County, Lu 'an City, Anhui Province*	1	1	1	0
Jinzhai County, Lu 'an City, Anhui Province*	14	13	0	1
Hanshan County, Maanshan City, Anhui Province	5	5	0	2
Chaoyang District, Beijing	20	13	0	3
Haidian District, Beijing	60	60	6	0
Huairou District, Beijing	8	8	2	0
Mentougou District, Beijing	50	50	2	0
Miyun District, Beijing	30	30	6	0
Pinggu District, Beijing	29	29	6	0
Shijingshan District, Beijing	50	50	6	0
Shunyi District, Beijing	27	27	5	1
Liangdang County, Longnan City, Gansu Province	20	20	7	1
Fuping County, Baoding City, Hebei Province	34	33	6	0
Xinglong County, Chengde City, Hebei Province	20	10	3	0
Haigang District, Qinhuangdao City, Hebei Province	18	9	2	0
Jingxing County,Shijiazhuang City, Hebei Province	20	20	6	0
Pingshan County, Shijiazhuang City, Hebei Province	38	30	5	0
Luoning County, Luoyang City, Henan Province	34	10	1	0
Nanle County, Puyang City, Henan Province	21	20	0	1
Gushi County, Xinyang City, Henan Province*	5	5	1	0
Guangshan County, Xinyang City, Henan Province*	3	3	0	3
Luoshan County, Xinyang City, Henan Province*	56	56	4	2
Pingqiao District, Xinyang City, Henan Province*	8	8	0	6
Shangcheng County, Xinyang City, Henan Province*	20	11	2	1
Xinxian County, Xinyang City, Henan Province*	14	6	0	6
Gongyi City, Zhengzhou City, Henan Province	7	7	2	0
Hongan County, Huanggang City, Hubei Province*	51	40	2	1
Luotian County, Huanggang City, Hubei Province*	8	6	5	0
Macheng City, Huanggang City, Hubei Province*	6	6	0	6
Danjiangkou City, Shiyan City, Hubei Province	11	1	1	0
Guangshui City, Suizhou City, Hubei Province*	20	20	0	2
Jiangxia District, Wuhan City, Hubei Province	13	10	6	0
Nanzhang County,Xiangyang City, Hubei Province	9	9	1	0
Guidong County, Chenzhou City, Hunan Province	2	1	0	1
Hunchun City, Yanbian Prefecture, Jilin Province	50	50	6	0
Xuyi County, Huai'an City, Jiangsu Province	86	50	0	4
Donghai County, Lianyungang City, Jiangsu Province	4	1	1	0
Jiangning District, Nanjing City, Jiangsu Province	12	12	1	0
Liuhe District, Nanjing city, Jiangsu Province	12	11	1	0
Wuzhong District, Suzhou City, Jiangsu Province	8	8	1	0
Yixing City, Wuxi City, Jiangsu Province	12	10	6	0
Suichuan County, Ji 'an City, Jiangxi Province	21	21	0	4
Xiuyan County anshan City Liaoning Province	14	10	5	0
Jinzhou District, Dalian City, Liaoning Province	32	22	6	0

Location	No. ticks identified	No. Asian longhorned ticks identified	No. bisexual Asian longhorned ticks	No. parthenogentic Asian longhorned ticks
Lvshunkou District, Dalian City, Liaoning Province	12	8	3	3
Zhuanghe City, Dalian City, Liaoning Province	13	12	1	5
Fengcheng City, Dandong City, Liaoning Province	27	27	6	0
Kuandian County, Dandong City, Liaoning Province	30	10	3	0
Penglai City, Yantai City,Shandong Province	31	10	0	3
Changdao County, Yantai City,Shandong Province†	16	16	1	2
High-tech District, Weihai City, Shandong Province	34	34	0	6
Qingzhou City, Weifang City, Shandong Province	50	50	1	2
Laizhou City, Yantai City, Shandong Province	3	1	1	0
Lingchuan County, Jincheng City, Shanxi Province	27	16	2	0
Dingxiang County, Xinzhou City, Shanxi Province	9	9	4	0
Jishan County, Yuncheng City, Shanxi Province	33	33	12	0
Mian County, Hanzhong City, Shaanxi Province	50	50	3	0
Fu County, Yan 'an City, Shaanxi Province	11	11	3	0
Ganquan County, Yan'an City, Shaanxi Province	4	3	2	0
Songjiang District, Shanghai	1	1	0	1
Cangxi County, Guangyuan City, Sichuan Province	60	58	3	2
Chaotian District, Guangyuan City, Sichuan Province	41	40	4	1
Jizhou District, Tianjin	25	25	6	0
Tengchong District, Baoshan City, Yunnan Province	8	8	0	2
Anji City, Huzhou City, Zhejiang Province	9	7	5	0
Dinghai District, Zhoushan City, Zhejiang Province	5	5	5	0
Daishan County, Zhoushan City, Zhejiang Province	82	25	1	0
Shengsi County, Zhoushan City, Zhejiang Province†	31	31	1	1
Kaizhou District, Chongqing	6	3	0	3

*Counties were located in Dabie Mountain.
†Parthenogenetic ticks were collected near the house of the patient who had severe fever with thrombocytopenia syndrome.

Appendix Table 2. Location and polyploid information for Asian longhorned tick samples

Location	No. Asian longhorned tick DNA samples	Ploidy
Oita Prefecture, Japan	3	Bisexual
Kagoshima Prefecture, Japan	5	Parthenogenetic
Jeju Province, South Korea	2	Parthenogenetic
New Jersey, USA	1	Parthenogenetic
New South Wales, Australia	2	Parthenogenetic
Queensland, Australia	2	Parthenogenetic
Okayama Prefecture, Japan	5	Parthenogenetic
South Island, New Zealand	1	Parthenogenetic

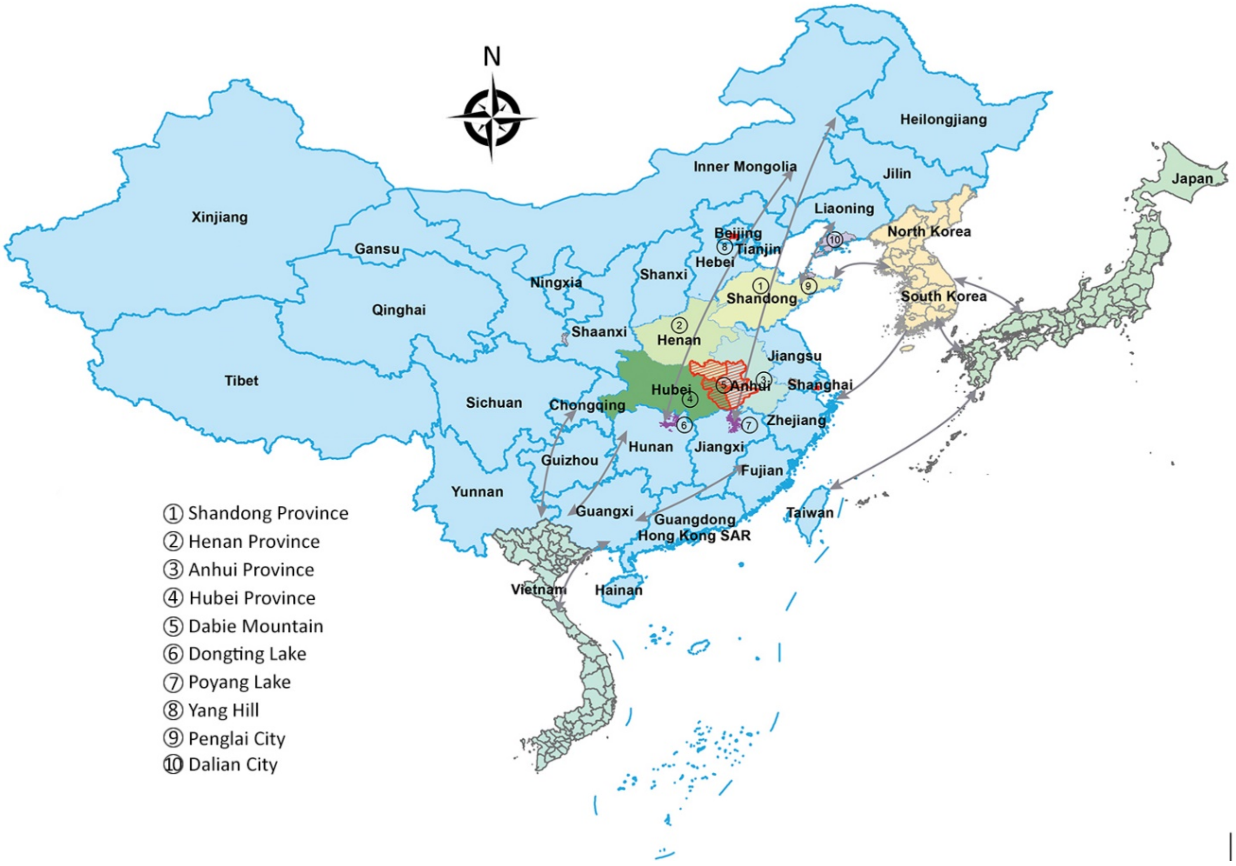

Appendix Figure 1. Locations analyzed in this study, eastern Asia. Arrows indicate bird migration routes.

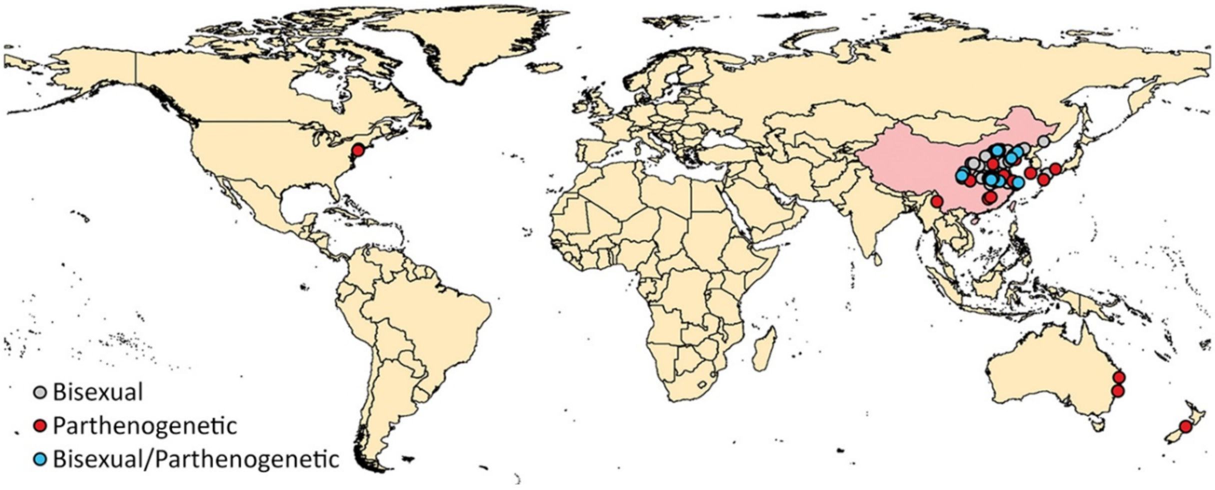

Appendix Figure 2. Bisexual and parthenogenetic Asian longhorned ticks collected in the Asia–Pacific area. Red dots indicate parthenogenetic ticks; gray dots indicate bisexual ticks, and blue dots indicate parthenogenetic and bisexual ticks.

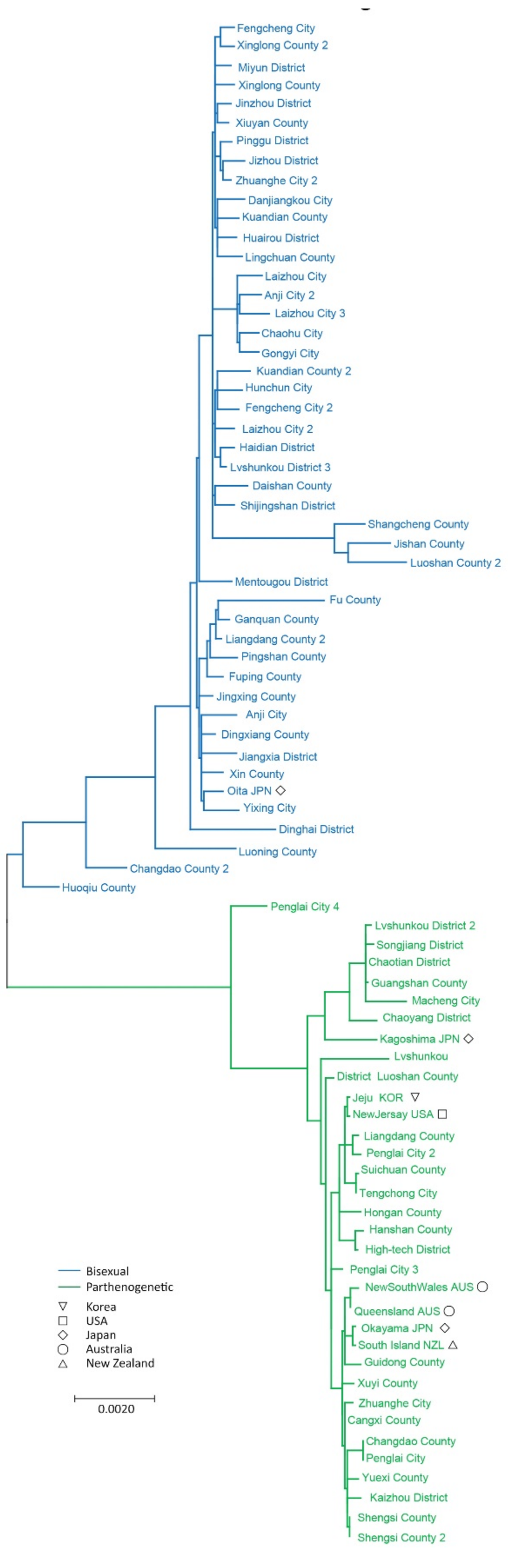

Appendix Figure 3. Maximum-likelihood tree established with the mitochondrial genomes of Asian longhorned ticks collected in the Asian–Pacific region. Multiple ticks from the same county are indicated by the numbers. Scale bar indicates nucleotide substitutions per site.

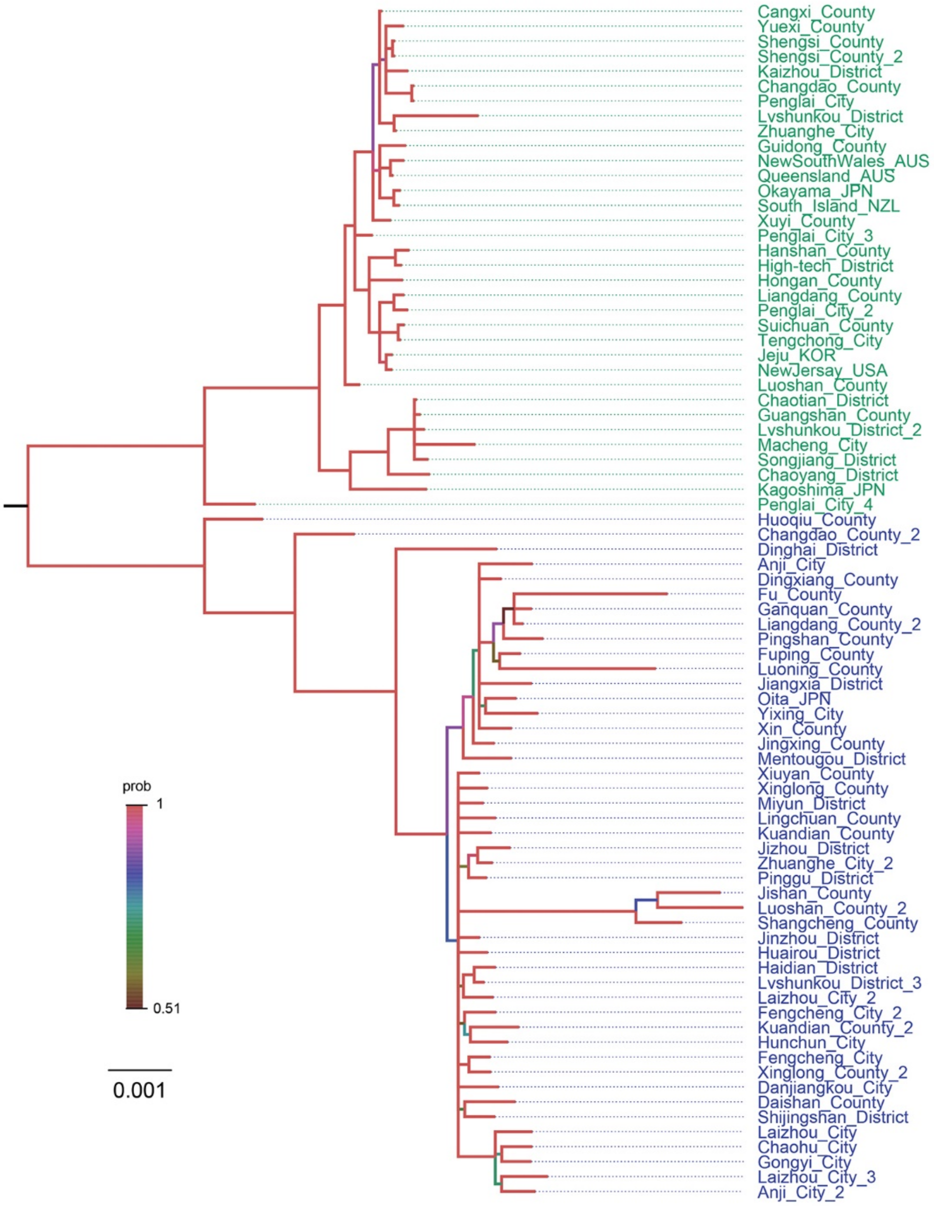

Appendix Figure 4. Bayesian phylogenetic tree of parthenogenetic (green) and bisexual (blue) Asian longhorned ticks. Colors of the branches indicate posterior probabilities. Scale bar indicates nucleotide substitutions per site. This tree was constructed by using MrBays-3.2.7 (http://nbisweden.github.io/MrBayes/index.html) with 1,500,000 generations, and the average SD of split frequencies of the tree is <0.01. prob, probability.

```
                              8490       8500
                                .          .
Chaohu City          AAGTTTTAATAAATAAATTAAATTATT
Gongyi City          AAGTTTTAATAAATAAATTAAATTATT
Huoqiu County        AAGTTTTAATAAATAAATTAAATTATT
Mentougou District   AAGTTTTAATAAATAAATTAAATTATT
Laizhou City         AAGTTTTAATAAATAAATTAAATTATT
Luoshan County       AAGTTTTAATAAA-AAATTAAATTATT
Chaoyang District    AAGTTTTAATAAA-AAATTAAATTATT
Shengsi County       AAGTTTAAATAAA-AAATTAAATTATT
Guidong County       AAGTTTAAATAAA-AAATTAAATTATT
Chaotian District    AAGTTTTAATAAA-AAATTAAATTATT
```

Appendix Figure 5. Mitochondrial genome alignment between parthenogenetic and bisexual Asian longhorned tick populations. The upper 5 sequences were from bisexual ticks, and the lower 5 sequences were from parthenogenetic ticks. Blue rectangle indicates nucleotides analyzed. Red dashes indicate nucleotide deletions.

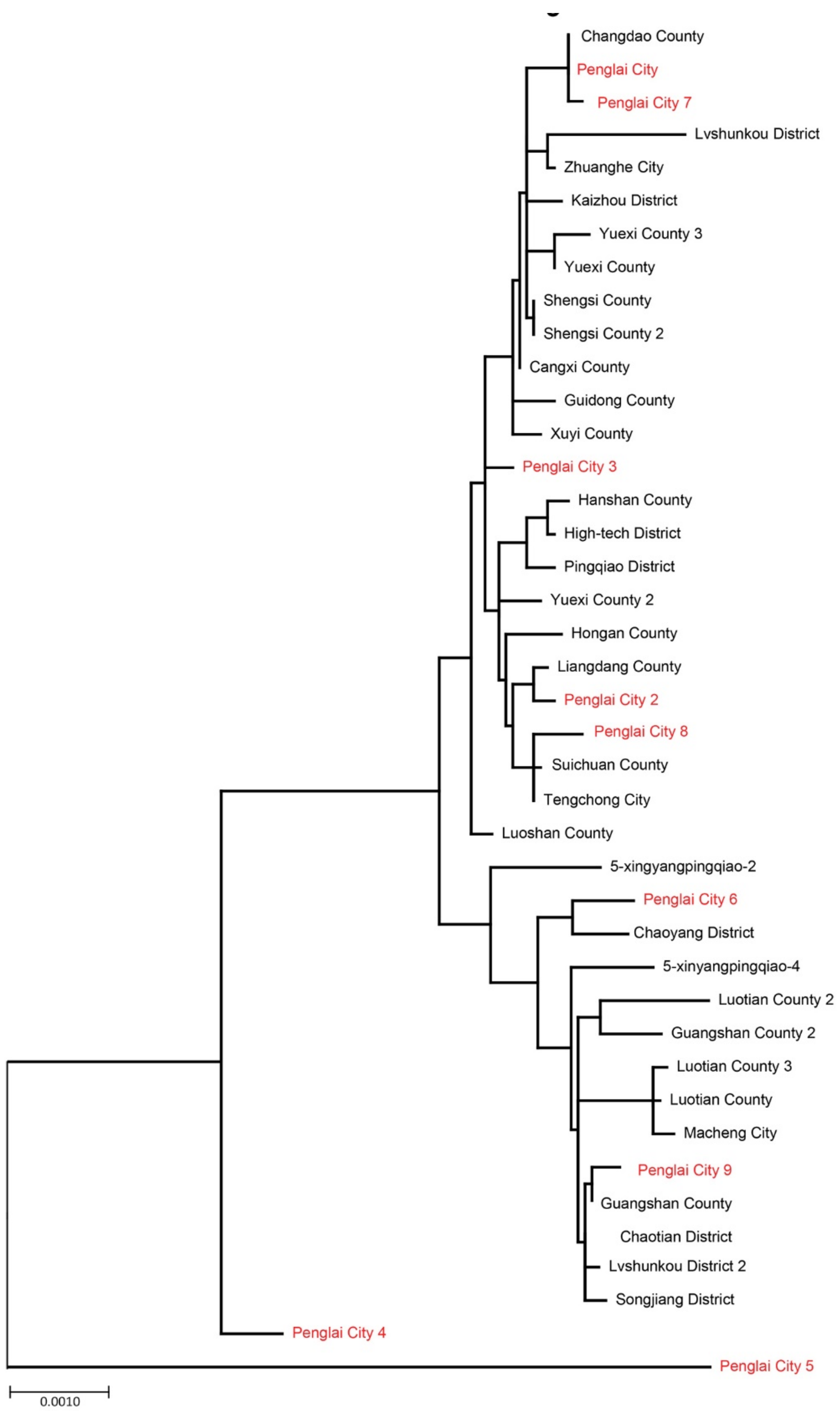

Appendix Figure 6. Phylogenetic analysis of parthenogenetic ticks collected from Penglai City (red) and samples from 15 provinces in China. Maximum-likelihood tree established with the mitochondrial genomes of ticks. Multiple ticks from the same county are indicated by numbers. Scale bar indicates nucleotide substitutions per site.
